# The enzymatic processing of α-dystroglycan by MMP-2 is controlled by two anchoring sites distinct from the active site

**DOI:** 10.1371/journal.pone.0192651

**Published:** 2018-02-15

**Authors:** Magda Gioia, Giovanni Francesco Fasciglione, Diego Sbardella, Francesca Sciandra, MariaLuisa Casella, Serena Camerini, Marco Crescenzi, Alessandro Gori, Umberto Tarantino, Paola Cozza, Andrea Brancaccio, Massimo Coletta, Manuela Bozzi

**Affiliations:** 1 Department of Clinical Sciences and Translational Medicine, University of Roma Tor Vergata, Roma, Italy; 2 CIRCMSB, Bari, Italy; 3 CNR Institute for Molecular Recognition, Roma Italy; 4 Istituto Superiore di Sanità, Roma, Italy; 5 CNR Institute for Molecular Recognition, Milano Italy; 6 School of Biochemistry, University of Bristol, Bristol, United Kingdom; 7 Institute of Biochemistry and Clinical Biochemistry, Catholic University, Roma Italy; Weizmann Institute of Science, ISRAEL

## Abstract

Dystroglycan (DG) is a membrane receptor, belonging to the dystrophin-glycoprotein complex (DGC) and formed by two subunits, α-dystroglycan (α-DG) and β-dystroglycan (β -DG). The C-terminal domain of α-DG and the N-terminal extracellular domain of β -DG are connected, providing a link between the extracellular matrix and the cytosol. Under pathological conditions, such as cancer and muscular dystrophies, DG may be the target of metalloproteinases MMP-2 and MMP-9, contributing to disease progression. Previously, we reported that the C-terminal domain α-DG (483–628) domain is particularly susceptible to the catalytic activity of MMP-2; here we show that the α-DG 621–628 region is required to carry out its complete digestion, suggesting that this portion may represent a MMP-2 anchoring site. Following this observation, we synthesized an α-DG based-peptide, spanning the (613–651) C-terminal region. The analysis of the kinetic and thermodynamic parameters of the whole and the isolated catalytic domain of MMP-2 (cdMMP-2) has shown its inhibitory properties, indicating the presence of (at least) two binding sites for the peptide, both located within the catalytic domain, only one of the two being topologically distinct from the catalytic active groove. However, the different behavior between whole MMP-2 and cdMMP-2 envisages the occurrence of an additional binding site for the peptide on the hemopexin-like domain of MMP-2. Interestingly, mass spectrometry analysis has shown that α-DG (613–651) peptide is cleavable even though it is a very poor substrate of MMP-2, a feature that renders this molecule a promising template for developing a selective MMP-2 inhibitor.

## Introduction

Dystroglycan (DG) is a membrane receptor belonging to the dystrophin-glycoprotein complex (DGC). DG is formed by two subunits, alpha-dystroglycan (α-DG) and beta-dystroglycan (β -DG), that provide a link between the extracellular matrix and the cytoskeleton [1]. α-DG is a highly glycosylated extracellular protein that interacts with laminin, agrin and other extracellular proteins [2, 3], whereas β -DG is a transmembrane protein, associated with actin through dystrophin [4]. Inside the cell, β -DG is involved in a network of interactions with many proteins, including some factors belonging to different signaling pathways [5, 6]. The two subunits are held together by non-covalent interactions occurring between the C-terminal domain of α-DG and the extracellular N-terminal domain of β -DG [7]. These interactions play a crucial role for the integrity of the entire DGC [8], that is in turn responsible for the stability of the plasma membrane, especially in the skeletal muscle and in the central nervous system, where DG is mainly expressed [9]. Under pathological conditions, such as cancer and neuromuscular diseases (including severe muscular dystrophies) [10], disruption of the DG subunits is often associated to the over-expression of some members of the metalloproteinase (MMP) family [11–15], which are Zn^2+^-dependent endopeptidases critical for tissue homeostasis and cell signaling [16, 17]. The structural modular architecture of MMP family shows (from N-terminus to C-terminus) a propeptide domain, which is removed upon enzyme activation, a catalytic domain and a hemopexin-like domain, which are connected by a hinge region [18]. The isolated catalytic domain of all MMP members retains the capability of cleaving linear peptidic substrates according to the specific substrate selectivity of each single subclass of the family [19]. Importantly, MMP gelatinases, namely MMP-2 and MMP-9, possess a unique additional collagen binding domain (CBD), inserted within the MMP’s catalytic domain sequence [20]. The peculiarity of the CBD domain concerns its involvement in the recognition and binding of protein substrates, a role exerted prevalently by the hemopexin-like domain in other MMPs, providing an ancillary binding surface which governs the cleavage specificity of very complex macromolecular substrates, such as 3D triple helical collagens [21–26].

Many physiological substrates for MMPs, including membrane proteins, are shed from the cell surface, often showing distinct biological functions in the proteolyzed form [19, 27, 28]. In particular, MMP gelatinases remove the extracellular N-terminal domain of β -DG, producing a 30 kDa truncated form of β -DG [11, 29, 30]. Two molecular mechanisms, differing between the two gelatinases, have been described *in vitro* for the shedding of the β -DG N-terminal domain. Thus, MMP-9 operates the first cleavage, removing at the N-terminal region of about 60 amino acids, which is further degraded, and leaving an intact C-terminal fragment of about 30 amino acids [31]; on the other hand, MMP-2 completely disrupts the β -DG N-terminal domain, producing multiple cleavages [32]. *In vivo*, disruption of the β -DG N-terminal domain is likely to leave the C-terminal domain of α-DG exposed to a variety of endopeptidases, that exert their catalytic activity within the extracellular matrix. However, recent findings report that double-targeting of MMP-2 and MMP-9 cannot prevent cleavage of β -DG in sarcoglycanopathy [33]. Processing and secretion of N-terminal domain of α-DG in cell culture media has been previously reported [34]. Further, *in vitro* we have revealed that native α-DG can be degraded by MMP-2, with the α-DG C-terminal domain representing the region most susceptible to the protease activity [35]. Computational analysis on all MMP cleavage sites reported in literature showed that MMPs cleave preferentially in exposed loops and in disordered regions of proteins [36]. No structural information is available for the α-DG C-terminal domain, but a modeling study suggested the presence of an Ig-like domain for the region approximately comprised between amino acids 500–600, followed by a coil-helix-coil motif spanning the amino acids 601–651 [37]. *In vitro*, the recombinant α-DG C-terminal domain can be degraded by MMP-2, whereas a shorter co-purified α-DG-fragment is fully resistant to the enzyme, suggesting the presence of a MMP-2 recognition site involving the missing amino acids [35]. In this work, we have assigned by mass spectrometry the sequence of this shorter fragment as α-DG (483–621), and we focused our investigation on the full C-term construct α-DG (483–628), following its proteolysis kinetics by MMP-2. Overall, we confirmed the proposed mechanism and showed that a peptide, spanning the last 39 amino acids of the α-DG C-terminus, namely α-DG (613–651) peptide, can modulate (already within the nanomolar range of concentrations) the MMP-2 catalysis of the entire enzyme and of its isolated catalytic domain (cdMMP-2).

## Materials and methods

### Materials

The purity of Human recombinant MMP-2 of whole proenzymes (R&D System, London, UK) and its catalytic domain cdMMP-2 (Biomol International) was measured by sodium dodecyl sulfate (SDS) polyacrylamide gel electrophoresis (PAGE) according to the Laemmli’s method. After the gels had been run, they were stained using a silver staining kit (Bio-Rad, Hercules, CA, USA). The broad spectrum protein markers (Bio-Rad, Hercules, CA, USA) were used as molecular weight standards. The stability of MMP-2 was checked by zymography (employing as the substrate either gelatin or type I collagen) [38]. The MMP-2 is perfectly stable (without undergoing any autocatalytic activity in the presence of the α-DG solution at 37°C) for well over 60 min, which covers by far the time period needed for the measurements of the catalytic parameters (*i*.*e*.,15 minutes).

p-Hydroxybenzyl alchol resin (Wang) and *N*-α-Fmoc-L-amino acids used during chain assembly were purchased from Iris Biotech GmbH (Marktredwitz, Germany). [2-(1*H*-benzotriazol-1-yl)-1,1,3,3-tetramethyluronium hexafluorophosphate] (HBTU) was purchased from Fluka (Buchs, Switzerland), *N*,*N’*-dimethylformamide (DMF) and trifluoroacetic acid (TFA) were from Carlo Erba (Rodano, Italy). *N*,*N’*-diisopropylethylamine (DIEA), dichloromethane (DCM), triisopropylsilane (TIPS), N-methyl pirrolidone (NMP) and all other organic reagents and solvents, unless stated otherwise, were purchased in high purity from Sigma-Aldrich (Steinheim, Germany). All solvents for solid-phase peptide synthesis (SPPS) were used without further purification. HPLC grade acetonitrile and ultrapure 18.2 Ω water (MilliQ) were used for the preparation of all solvents for liquid chromatography.

### DNA manipulation

The full-length cDNA encoding for murine DG was used as a template to generate by PCR two DNA construct corresponding to the C-terminal region of α-DG, α-DG(483–628). Appropriate primers were used to amplify the DNA sequence of α-DG(483–628): forward 5’-CCC**GTCGAC**AGTGGAGTGCCCCGTGGGGGAGAAC-3’ and reverse 5’-CCC**GAATTC**TTATACCAAAGCAATTTTTCTTGTGAATG-3’, *SalI* and *EcoRI* restriction sites are in bold type.

### Protein expression and purification

The DNA construct obtained was purified and cloned into a bacterial vector which is appropriate to express the protein as thioredoxin fusion product, also containing an N-terminal 6His tag and a thrombin cleavage site. The recombinant fusion protein was expressed in *Escherichia coli* BL21(DE3) Codon Plus RIL strain (Agilent Technologies, Australia), since no glycosylation sites have been found in the native eukaryotic protein [39], and purified using nickel affinity chromatography. The construct of interest was obtained upon thrombin cleavage. Tricine/SDS-PAGE was used to check the purity of the recombinant proteins under analysis.

### Chemical synthesis and purification of the C-term peptide

Linear peptide was assembled on a Wang resin (0,6 mmol/g) in a 0.10 mmol scale. Resin was properly swelled prior use with a NMP/DCM mixture. Peptide assembling was performed automatically on a Biotage ALSTRA peptide synthesizer using a Fmoc-chemistry protocol. Coupling of entering Fmoc-protected amino acids was performed using HBTU/DIEA (1:1:2, 4 equivalents excess over resin loading, 1 x 5min, 75 C). Deprotection steps were performed by treatment with a 20% piperidine solution in DMF (2x5min, RT). Following each coupling or deprotection step, resin-bound peptide was washed 5 times with DMF. Following chain assembly, peptides were cleaved from the resin using a TFA 90%, Water 5%, Phenol 2.5%, TIPS 2.5% mixture (2 hours, RT). Following precipitation in cold diethyl ether, crude peptides were collected by centrifugation and subsequently washed with further cold diethyl ether to remove scavengers. Peptides were then dissolved in a 50% aqueous acetonitrile 0.07% TFA buffer and underwent RP-HPLC purification.

Analytical and semi-preparative Reversed Phase High Performance Liquid Chromatography (RP-HPLC) were carried out on a Tri Rotar-VI HPLC system equipped with a MD-910 multichannel detector for analytical purposes or with a Uvidec-100-VI variable UV detector for preparative purpose (all from JASCO, Tokyo, Japan). A Phenomenex Jupiter 5μ C18 90Å column (150 x 4.6 mm) was used for analytical runs and a Phenomenex Jupiter 10μ C18 90Å (250 x 21.2 mm) for peptide purification. Data were recorded and processed with Borwin software. A linear gradient of eluent B (eluent A = H2O/ 3% CH_3_CN / 0.07% TFA, eluent B = 70% CH_3_CN/ 30% H2O/ 0.07% TFA) was employed at a flow rate of 1mL/min for analytic purposes. UV detection was recorded in the 220–320 nm range. Purification of the peptides was achieved by preparative RP-HPLC at a flow rate of 14 mL/min using a linear gradient of eluent B. Pure RP-HPLC fractions (>95%) were combined and lyophilized. Mass spectra were collected separately.

### Mass spectrometry analysis of the recombinant a-DG(483–628) domain and of the α-DG(613–651) peptide

Recombinant α-DG (483–628) domain was separated on 1D-gel NuPAGE 4–12% (Novex, Invitrogen) and stained with the Colloidal Blue Staining kit (Invitrogen). The stained bands were cut, incubated with 10 mM DTT for 1h at 56°C, then with 50 mM iodoacetamide for 45min at RT in the dark, subsequently dried before enzymatic digestion that was performed with 12.5 ng/ml LysC or GluC (Roche) at 37°C over night.

Samples containing the (613–651) peptide were cleaned by 30kDa cutoff filters to remove MMP-2 and desalted by C18 Zip Tip (Millipore) before mass spectrometry analysis. Peptide mixture deriving from the enzymatic reactions (α-DG (483–628) domain treated with LysC, GluC or (613–651) peptide incubated with MMP2) were analyzed by nanoflow-reversed-phase liquid chromatography tandem mass spectrometry (RP-LC-MS/MS) using an HPLC Ultimate 3000 (DIONEX, Sunnyvale, CA U.S.A) connected on line with a linear Ion Trap (LTQ, Thermo, San Jose, CA). Peptides were desalted in a trap-column (AcclaimPepMap100 C18, LC Packings, DIONEX) and then separated in a 10 cm long fused silica capillary (SilicaTipsFS 360-75-8, New Objective, Woburn, MA, USA), slurry-packed in-house with 5 μm, 200 Å pore size C18 resin (Michrom BioResources, CA). Peptides were eluted using a 45 min long linear gradient from 20% to 50% acetonitrile in presence of 0.1% formic acid at 300 nl/min flow rate, followed by 50 min during which the column was washed and then equilibrated again. Spectra were acquired in positive ion mode (HV Potential 1.7–1.8kV) in a data-dependent mode: each full MS spectrum was followed by the fragmentation of the five most abundant precursor ions. MS/MS spectra were analyzed using the Proteome Discoverer 1.4 software (Thermo) and database from Swiss-Prot containing E. coli proteins and / or α-DG sequence.

### Enzymatic assays

Human MMP-2 proenzyme 2 was activated by incubating 0.1 mg/ml progelatinase solution with 0.25 mM aminophenyl mercuric acid (Sigma, St. Louis, MO, USA) at 37°C for 30 minutes. Whereas human recombinant cdMMP-2 human was dissolved in a solution of 50 mM Tris/HCl (pH 7.2), 0.1M NaCl and 10 mMCaCl. The actual concentration of active MMP-2 (i.e. whole MMP2 and cdMMP2) was determined by the classic fluorimetric assay [38], following the progressive decrease of hydrolysis (upon addition of an irreversible inhibitor, ilomastat (GM 6001) which stoichiometrically inhibits MMPs) of the MCA- Pro-Leu-Gly-Leu-DPA-Ala-Arg-NH_2_ fluorogenic substrate (λ_exc_ = 325 nm, λ_em_ = 398 nm). All measurements were performed at 37°C using a solution of 50 mM Tris–HCl, 0.1 M NaCl, 10 mM CaCl_2_ and 0.05% Brij 35 buffered at pH 7.3.

### α-DG domain degradation assay

The α-DG domain degradation by MMP-2 was performed in 50 mM Tris–HCl, 0.1 M NaCl, 10 mM CaCl_2_ the mixtures were kept at 37°C and small aliquots were harvested at different time intervals (ranging from 0 and 18 hours). The reactions were stopped by the addition of SDS-PAGE loading buffer containing 20 mM EDTA, boiled for 2 minutes and frozen to -80°C until they were used.

Equal aliquots from the incubation mixtures were collected at increasing times, the samples were separated on gradient precast TRIS glycine 10–20% SDS-PAGE gels and these were stained using 0.5% Commassie blue, followed by distaining until substrate bands were clearly visible. The Coomassie blue electrophoretic spots corresponding to each time intervals were analyzed by image analysis software (Image Quant TL, Amersham Biosciences). For the evaluation of pixel intensity, the pixel brightness through the region of interest was measured and plotted using Graphpad Prism v6 software. Histograms reported the relative amount intensity (expressed as arbitrary units and converted to percentage according to the ratio = band intensity at the starting point t = 0/ intensity at any given time interval). Data were presented as means± standard deviation of three independent experiments. The difference between the two groups was compared with one-way ANOVA followed by Tukey post-hoc test by GraphPad Prism, version 6.0 GraphPad software (La Jolla Ca), * p< 0.05 was considered significant.

### Modulation of the α-DG C-terminal peptide on the MMP-2 activity

Different concentrations of the α-DG peptide (*i*.*e*., final concentration range spanning between 0 μM and 4 μM) were incubated with 0.07 μM active MMP-2 for 30 min at 37°C to allow the interaction to occur. In the case of the cdMMP2 the kinetics were performed employing 0.06 μM cdMMP-2 at different concentration of α-DG (613–651) peptide in the range 0–2 μM. Different concentrations of MCA fluorogenic peptide (i.e: from 5 to 15 mM) were then added to this solution. The enzymatic reaction was recorded for 30 minutes at 37°C. The initial velocities were derived from the slopes within the first 15 minutes, which is the time period during which the rate is constant and less than 10% of the substrate was degraded. This ensured a steady-state condition for the first cleavage step, and it was a prerequisite for the subsequent analysis steps.

## Results

### Characterization of recombinant α-DG (483–628) domain by mass spectrometry

As already shown in our previous work [35], the *murine* α-DG C-term domain (construct expressed and purified in *E*. *coli*) displayed two electrophoretic bands: an upper band (~17 kDa) and a lower band (~ 15 kDa).

**Fig 1:**
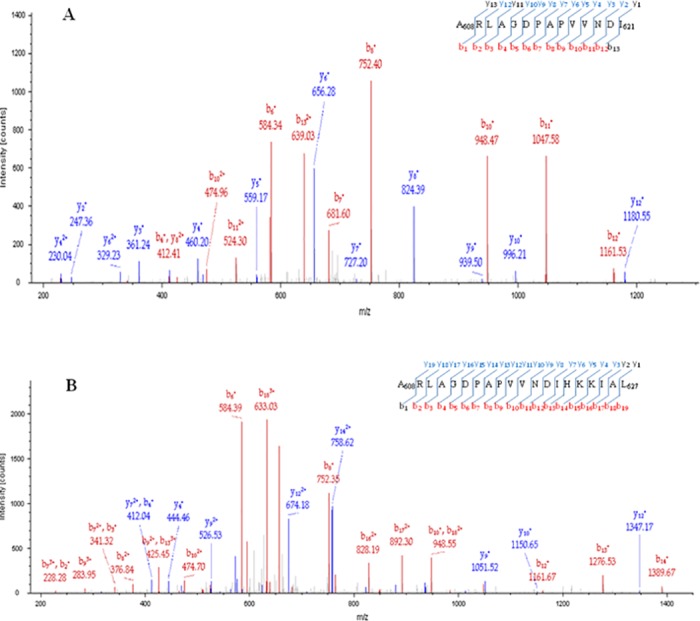
MS LC-MS/MS analysis of the purified αDG (483–628) domain. MS/MS spectra of the (608–621) peptide (A) and (608–627) peptide (B) deriving from LysC digestion of lower and upper band of the gel shown in Fig 2, respectively. Matched b and y ions are colored in red and blue, respectively.

To verify the absence of contamination from *E*. *coli* and confirm the identity of the purified α-DG (483–628) domain, the two bands have been enzymatically *in gel*-digested and analyzed by LC-MS/MS. The analysis of both bands confirmed the presence of several peptides deriving from the α-DG (483–628) domain and no contaminant proteins from *E*. *coli* were detected. Moreover, whilst the peptide mixture from the upper band allowed to map the sequence until the leucine at position 627, as revealed by the (608–627) peptide (Table 1 and Fig 1), the enzymatic digestion of the lower band produced the (608–621) peptide (Table 1 and Fig 1), which is absent in the upper band. These data indeed suggest that the lower band is a degradation product which co-purifies with the recombinant α-DG (483–628) domain, likely ending with the isoleucine at position 621.

**Table 1:** X Corr indicates cross-correlation score that is the sequest measure of the goodness of fit of experimental peptide fragments to theoretical spectra. Upper band and Lower band refer to the bands visible in the gel in the Fig 2. z is the peptide charge detected.

Position	Enzyme	Peptide sequence	Upper band		Lower Band	
			X Corr	z	X Corr	z
497–513	GluC	LKNHIDRVDAWVGTYFE	4.91	2	4.74	2
499–515	LysC	NHIDRVDAWVGTYFEVK	4.83	2	4.49	2
499–531	LysC	NHIDRVDAWVGTYFEVKIPSDTFYDNEDTTTDK	5.11	5	5.31	5
513–540	GluC	VKIPSDTFYDNEDTTTDKLKLTLKLRE	4.68	4	4.18	3
516–531	LysC	IPSDTFYDNEDTTTDK	3.7	2	3.44	2
516–533	LysC	IPSDTFYDNEDTTTDKLK	4.49	4	3.85	3
516–537	LysC	IPSDTFYDNEDTTTDKLKLTLK	3.99	5	4.5	3
532–547	LysC	LKLTLKLREQQLVGEK	4.01	4	3.04	3
541–572	GluC	QQLVGEKSWVQFNSNSQLMYGLPDSSHVGKHE	3.38	2	3.13	4
547–572	GluC	KSWVQFNSNSQLmYGLPDSSHVGKHE	6.81	3	7.17	3
547–572	GluC	KSWVQFNSNSQLMYGLPDSSHVGKHE	6.49	3	6.84	3
548–570	LysC	SWVQFNSNSQLmYGLPDSSHVGK	5.32	3	5.06	3
548–570	LysC	SWVQFNSNSQLMYGLPDSSHVGK	4.93	5	4.85	3
548–580	LysC	SWVQFNSNSQLMYGLPDSSHVGKHEYFMHATDK	3.2	2	3.05	5
573–590	GluC	YFmHATDKGGLSAVDAFE	4.98	2	4.19	2
573–590	GluC	YFMHATDKGGLSAVDAFE	4.37	4	3.92	2
581–595	LysC	GGLSAVDAFEIHVHK	4.51	2	4.73	2
608–621	LysC	ARLAGDPAPVVNDI			3.75	2
608–627	LysC	ARLAGDPAPVVNDIHKKIAL	4.65	2		

### The α-DG (613–651) peptide inhibits MMP-2 processing of α-DG (483–628) domain

**Fig 2:**
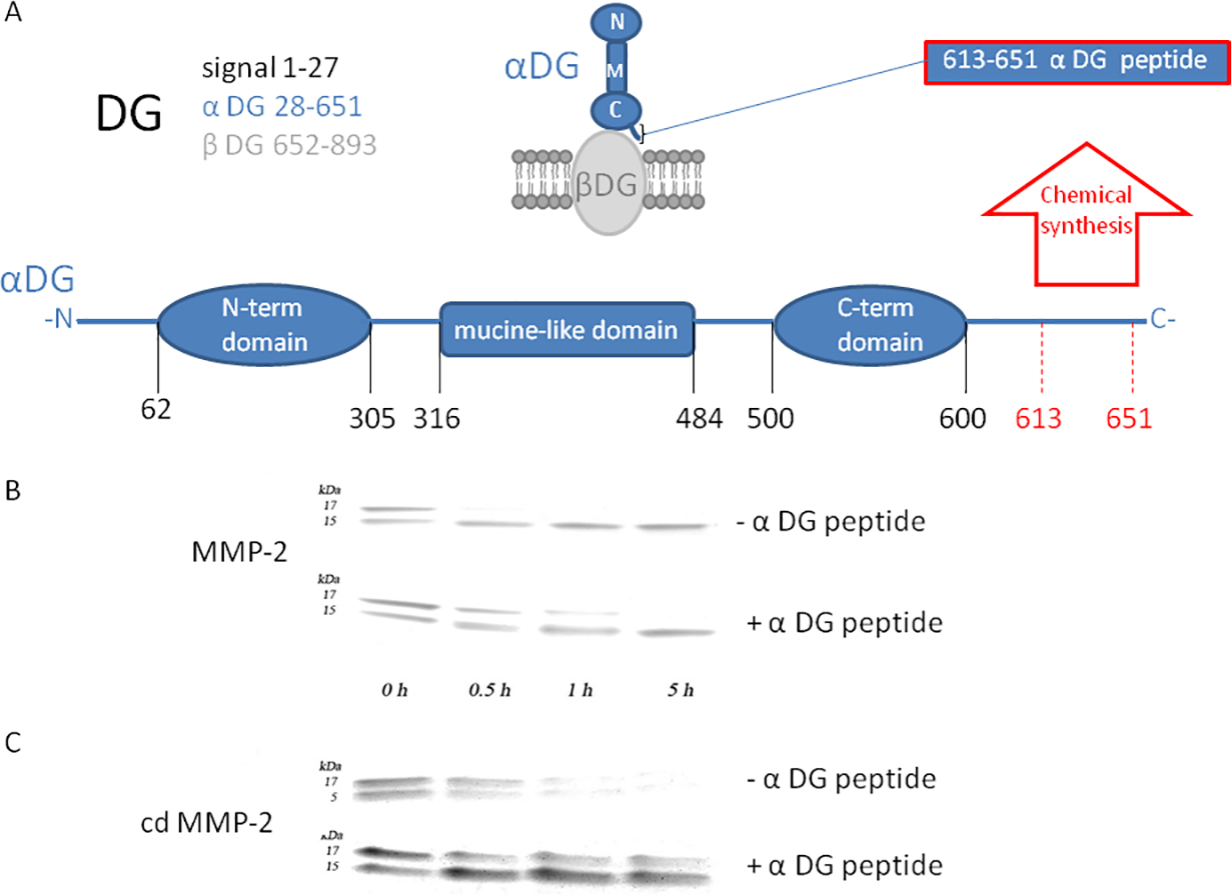
**The αDG (613–651) peptide affects the degradation of αDG (483–628) peptide** (*Panel A*) Aminoacidic sequences associated with each subunits of murine DG and its schematic representation. Enzymatic proteolysis profiles of the purified α-DG C-terminal domain (483–628) construct by MMP-2 (*Panel B*), or by cdMMP-2 (*Panel C*) are represented by the sodium dodecyl sulfate polyacrylamide gels stained with Coomassie blue. Four-point time course was represented: time 0, time 30 s, time 1 hour, time 5 hours in the absence (upper panel) and in the presence (lower panel) of 4 μM of the α-DG (613–651) peptide. Equal aliquots from the incubation mixtures were collected at increasing times, the reaction was stopped and samples were separated by TRIS glycine 10–20% precast gel.

Degradation kinetics of 7 μM α-DG (483–628) domain (see Fig 2) by 14 nM MMP-2 is analyzed by SDS-PAGE electrophoresis separation (Fig 2B, *upper lanes*). In line with our previous work [35], the intensity of the ~ 17 kDa electrophoresis band progressively reduces its intensity over time while, concurrently, the lower band (~ 15 kDa) is resistant to the MMP-2 proteolytic activity (Fig 2B, *upper lanes*). In order to unravel the role of the MMP-2 hemopexin-like domain for the differential fragmentation, the proteolysis kinetics of the α-DG (483–628) domain was also performed employing 30 nM of the isolated catalytic domain cdMMP-2. Fig 2C (*upper lanes*) shows that also the catalytic domain of MMP-2 (without the hemopexin-like domain) retains the capability of degrading the α-DG (483–628) domain, even though in this case the proteolytic cleavage of the α-DG domain appears less specific, the degradation occurring both on the 17 kDa band and the 15 kDa band (Fig 2C, *upper lanes*). Therefore, the data reported in Fig 2 clearly indicate that

1. the cleavage of the α-DG (483–628) domain by MMP-2 does not require the presence of the hemopexin-like domain, since also cdMMP-2 enzymatically processes this domain;
2. the hemopexin-like domain likely plays a functional regulatory role by addressing MMP-2 toward a specific binding site, located within the C-terminal region between amino acid positions 621–628, thus avoiding unspecific and widespread fragmentation of the all macromolecular substrate.
3. The presence of the hemopexin-like domain in the whole MMP-2 protects the α-DG 483–621 domain from the enzymatic processing.

Therefore, the hemopexin-like domain, though not required for the cleavage of the α-DG (483–628) domain, is nonetheless crucial for proper substrate positioning of MMP-2, envisaging its interaction with the 621–628 amino acid region.

**Fig 3:**
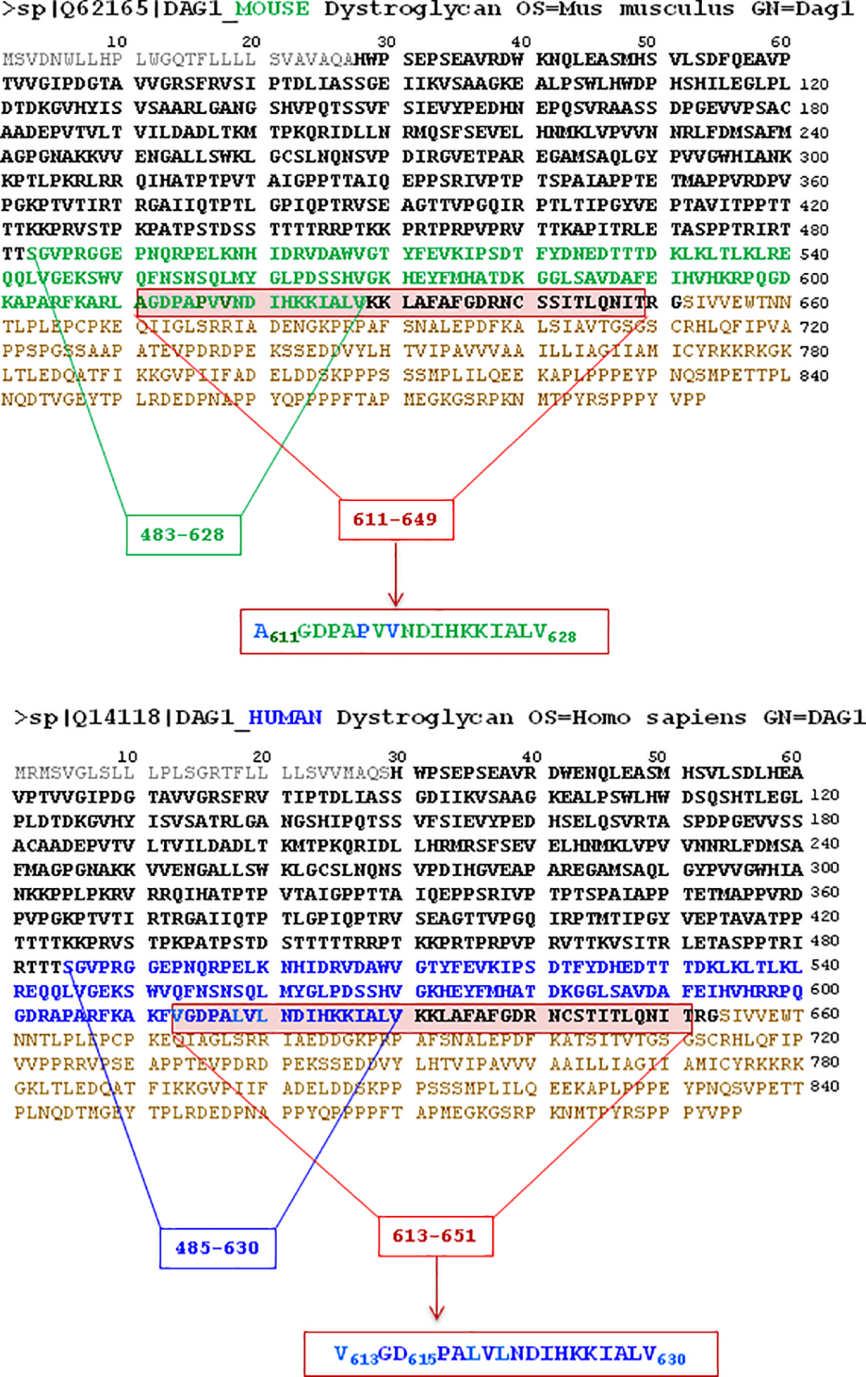
Comparison between the amino acid sequence of *murine* (*upper panel*) and *human* (*lower panel*) DG (according to Swiss-Prot database). Amino acid sequences for signal peptides are in gray, for α-DG in bold, and for α-DG in brown. Amino acid sequence of murine α-DG(483–628) are in bold green (*upper panel*) while the corresponding sequence of human α-DG(485–630) is in bold blue. The amino acid sequence of the peptide derived from the C-terminal portion of human α-DG (in the highlighted red rectangle), contains the sequence (613–630) that overlaps with the corresponding mouse α-DG sequence (611–628), except for amino acids in light blue in the red rectangle in lower panel.

In order to cast some light on the role of the 621–628 amino acid region on the α-DG processing, we have chemically synthesized a human α-DG (613–651) peptide which shares a closely similar amino acid sequence with murine α-DG 611–649 (see Fig 3). Therefore, we have investigated the functional effect of the α-DG (613–651) peptide on the enzymatic processing by MMP-2 of the α-DG (483–628) domain.

Under the same experimental conditions, the degradation rate of the α-DG (483–628) domain was much slower in the presence of 4 μM of α-DG peptide (Fig 2). Indeed, a significant decrease of the 17 kDa band could be observed only after 5 hours of MMP-2 activity (Fig 2A, *lower lanes*) and a similar inhibitory effect was also detected in the case of cdMMP-2 (Fig 2B, *lower lanes*); furthermore, in the case of cdMMP-2, the presence of the α-DG (613–651) peptide appears to inhibit even the enzymatic processing of the 15 kDa band (Fig 2B, *lower lanes*). This feature suggests that the inhibitory effect is exerted through a direct binding of the α-DG (613–651) peptide on cdMMP-2. This interaction not only interferes with the proteolytic cleavage of the α-DG (613–651) sequence, but it also impairs the interaction of cdMMP-2 with additional substrate exosites, which can explain the extended and widespread fragmentation of the shorter α-DG (483–621) domain in the absence of the peptide (Fig 2B, *upper lanes*).

The similar inhibitory behavior suggests that the binding site of the α-DG (613–651) peptide is located at the catalytic domain of MMP-2 and, given that the α-DG(483–628) domain partially overlaps with the α-DG(613–651) peptide, this result indicates also that the interaction of α-DG(483–628) domain with MMP-2 involves also the catalytic domain.

### MMP-2 enzymatic activity toward a fluorogenic peptide is inhibited by the human α-DG C-term (613–651) peptide

**Fig 4:**
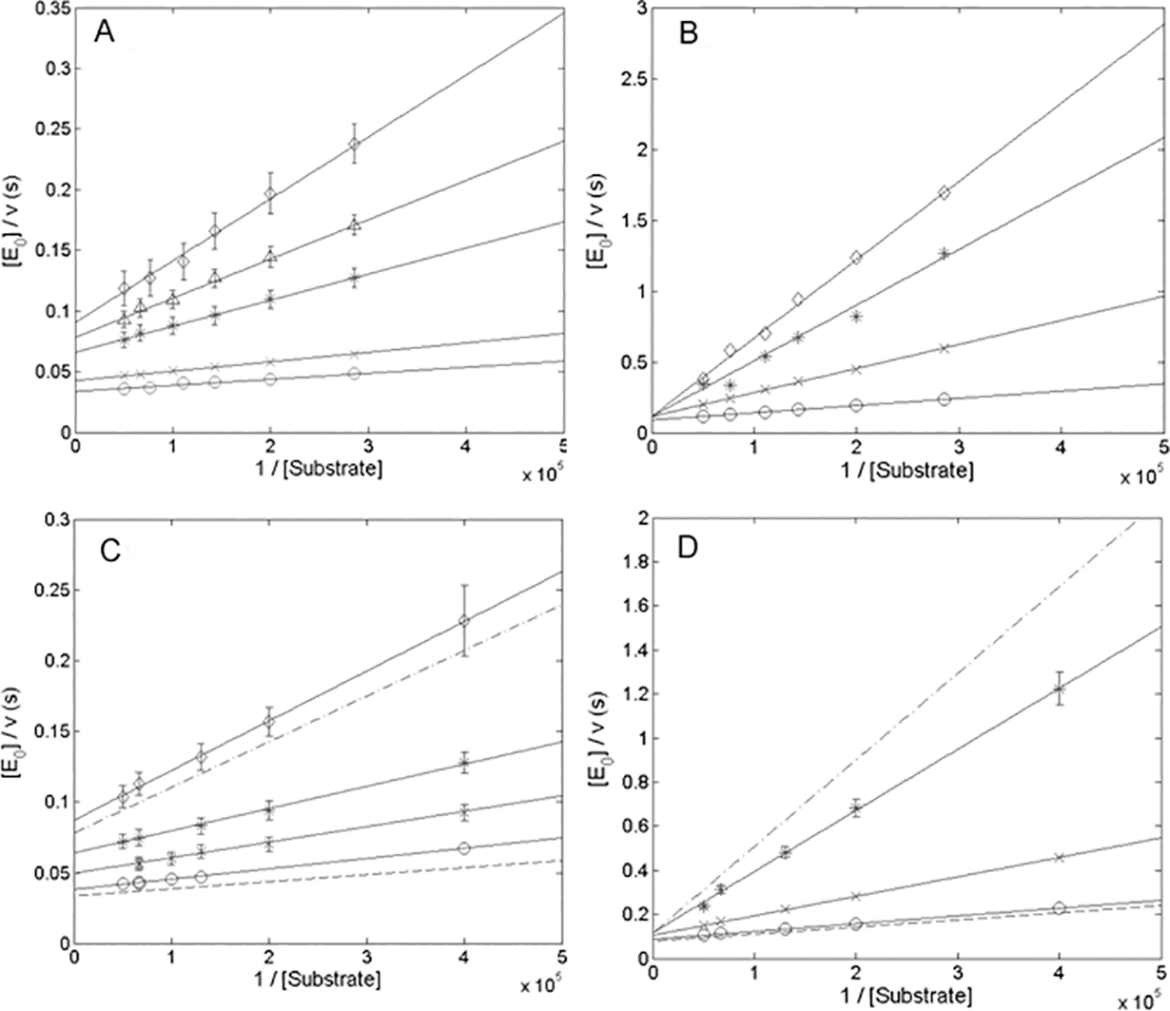
The effect of αDG (613–651) peptide on the catalytic parameters for the MMP-2 proteolysis. Lineweaver-Burk plot of the enzymatic activity of 60 nM MMP-2 at 37°C at pH 7.3 as a function of the fluorogenic substrate concentration at different concentrations of α-DG(613–651) peptide, namely (*panel A*) 0 (o), 23 nM (x), 125 nM (*), 200 nM (Δ) and 0.4,μM (25CA), and (*panel B*) 0.4 μM (o), 1 μM (x), 2 μM (*) and 4 μM (◊). Continuous lines have been obtained by applying Eqs. (1)–(3), employing parameters reported in Table 2. (*Panel C*): namely 0 (o), 23 nM (x), 60 nM (*) and 0.2 μM (◊), and, (*panel D*) 0.2 μM (o), 0.6 μM (x) and 2 μM (*) and, (*panel D*) 0.2 μM (o), 0.6 μM (x) and 2 μM (*). Continuous lines have been obtained by applying Eqs. (1)–(3), employing parameters reported in Table 2. Dashed line corresponds to the Lineweaver-Burk plot of the enzymatic activity of whole MMP-2 in the absence. Dashed-dotted lines corresponds to the Lineweaver-Burk plot in the presence of the α-DG(613–651) peptide 0.2 μM and 2 μM α-DG(613–651) peptide in *panel C* and *panel D*, respectively. Where not shown, standard deviation is smaller than symbol.

In order to determine the inhibitory mechanism, exerted by the α-DG (613–651) peptide on MMP-2 and its cd-MMP-2, a small fluorogenic substrate (MCA-Pro-Leu-Gly-Leu-DPA-Ala-Arg-NH_2_) was employed. Fig 4 reports the Lineweaver-Burk plots of the degradation kinetics of the fluorogenic substrate carried out by the whole MMP-2 in the presence of increasing amounts of the α-DG(613–651) peptide over a range of concentrations spanning between 0 and 4 μM (Fig 4A and 4B). It immediately comes out that the inhibitory effect displays a bimodal behavior, since at lower concentrations (*i*.*e*., between 23 nM and 0.4 μM) a non-competitive mechanism seems operative (see Fig 4A), while at higher concentrations a competitive inhibition comes into play (see Fig 4B). Such a bimodal behavior can be observed also in Fig 4C and 4D, which represents the Lineweaver-Burk plots of the degradation kinetics of the fluorogenic substrate carried out by cdMMP-2 in the presence of increasing amounts of the α-DG(613–651) peptide, whose concentration values ranged between 0 and 2 μM (Fig 4C and 4D).

**Fig 5:**
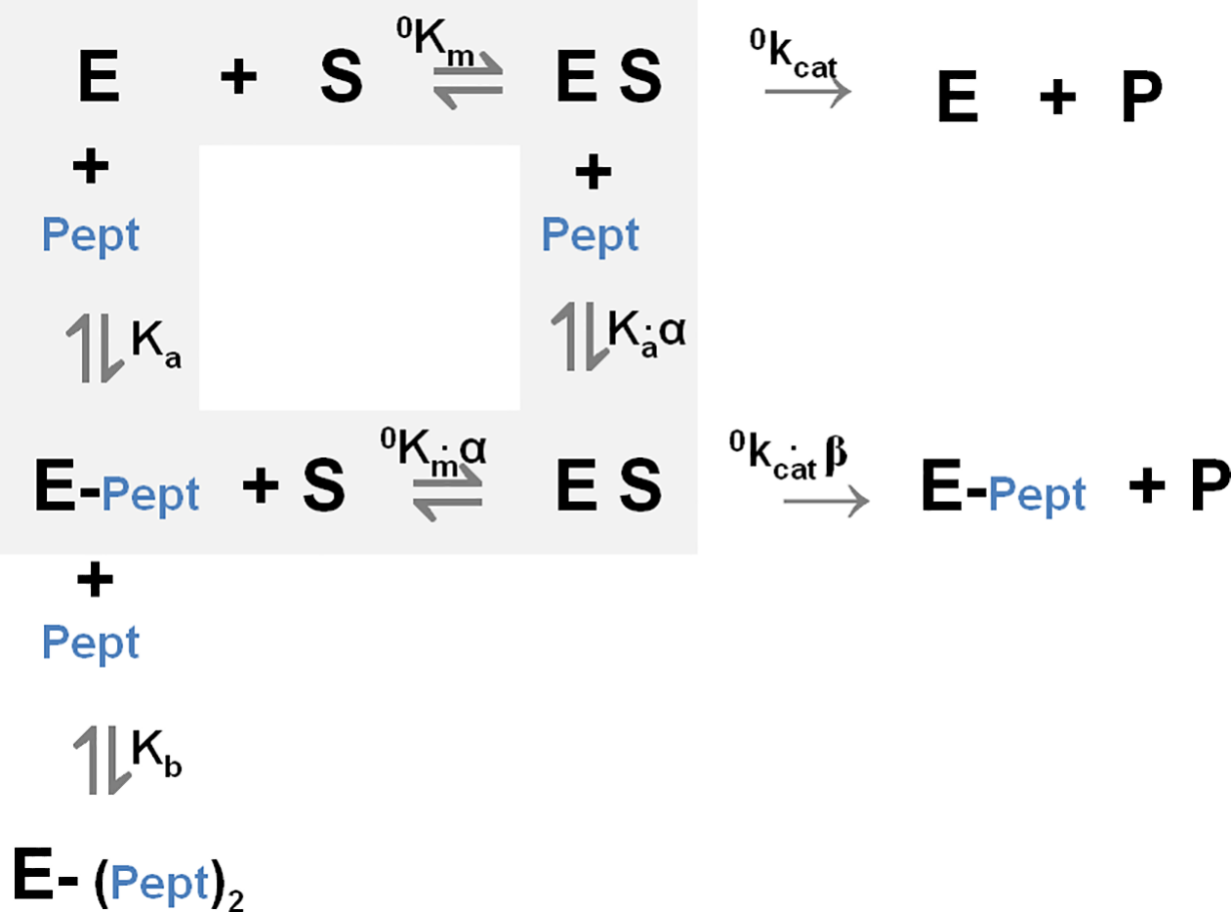
Thermodynamic and kinetic scheme for the effect of the αDG(613–651) peptide on the catalytic processing of the fluorogenic peptide. Where, pept represents the αDG(613–651) peptide, ^*0*^*k*_*cat*_ and ^*0*^*K*_*m*_ are the observed catalytic parameters in the absence of the peptide, *K*_*a*_ and *K*_*b*_ are the peptide dissociation constants to the free enzyme for the two binding sites, *α* and *β* are the interaction parameters.

This behavior, which envisages the presence of (at least) two binding sites for the α-DG (613–651) peptide on the catalytic domain of MMP-2, can be kinetically and thermodynamically described by the scheme reported on Fig 5; where, ^*0*^*k*_*cat*_ and ^*0*^*K*_*m*_ are the observed catalytic parameters in the absence of the peptide, *K*_*a*_ and *K*_*b*_ are the peptide dissociation constants to the free enzyme for the two binding sites, *α* is the interaction parameter, which quantifies the effect on *K*_*m*_ of the α-DG(613–651) peptide binding to the first binding site (α > 1 indicates a negative effect with a decreased affinity, α < 1 a positive effect and α = 1 no effect), *β* is the interaction parameter which estimates the effect on *k*_*cat*_ of the binding of α-DG(613–651) peptide to the first binding site (β > 1 indicates a positive effect with an increased rate constant, β < 1 a negative effect and β = 1 no effect).

Data reported in Figs 4 and 5 have been analyzed according to the following equation:

$$\frac{\left[E_{0}\right]}{v}=\frac{{}^{obs}K{}_{m}}{{}^{obs}k{}_{cat}}\cdot \frac{1}{\left[S\right]}+\frac{1}{{}^{obs}k{}_{cat}}$$ (Eq 1)

where [*E*_0_] is the total enzyme concentration, *ν* is the observed velocity (expressed as mol/s), [*S*] is the substrate concentration, ^*obs*^*k*_*cat*_ and ^*obs*^*K*_*m*_ are the observed catalytic parameters, namely

$${}^{obs}k{}_{cat}={}^{0}k{}_{cat}\cdot \frac{\left(K_{a}\cdot \alpha +\beta \cdot \left[Pept\right]\right)}{\left(K_{a}\cdot \alpha +\left[Pept\right]\right)}$$ (Eq 2)

and

$${}^{obs}K{}_{m}={}^{0}K{}_{m}\cdot \frac{\alpha \cdot (K_{a}\cdot K_{b}+K_{b}\cdot \left[Pept\right]+{\left[Pept\right]}^{2})}{K_{b}\cdot (K_{a}\cdot \alpha +\left[Pept\right])}$$ (Eq 3)

where ^*0*^*k*_*cat*_ and ^*0*^*K*_*m*_ are the catalytic paramenters in the absence of peptide and all other symbols have the same meaning as described above.

**Fig 6:**
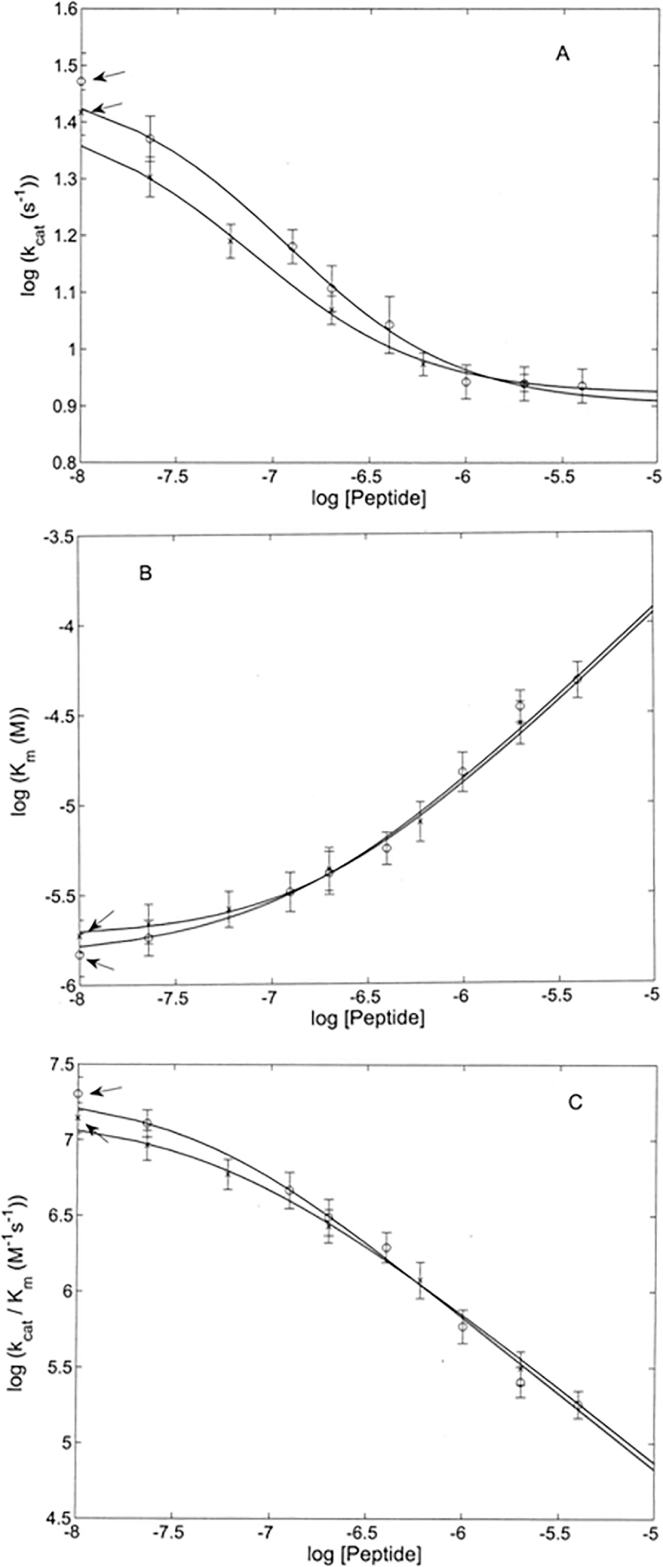
The αDG (613–651) peptide modulation of MMP-2 catalysis. Dependence on the α-DG(613–651) peptide concentration of *k*_*cat*_ (*panel A*), *K*_*m*_ (*panel B*) and *k*_*cat*_*/K*_*m*_ (*panel C*) for the enzymatic processing of the fluorogenic peptide by whole MMP-2 (o) and by cdMMP-2 (x) at pH 7.3 and 37°C, as obtained by the analysis of data reported in Figs 4 and 5. Continuous lines have been obtained by applying Eqs (2) and (3), employing parameters reported in Table 1. Arrows indicate the value of the parameter in the absence of the α-DG(613–651) peptide, which is represented as “o” for the intact MMP-2 and as “x” for cdMMP-2.

The global fitting of data, reported in Fig 4, employing simultaneously Eqs (1), (2) and (3), allowed to obtain all parameters reported in Table 2. The peptide concentration dependence of catalytic parameters (*k*_*cat*_ and *K*_*m*_) for both MMP-2 and cdMMP-2 confirms the appropriateness of the scheme adopted (Figs 5 and 6, see Table 2). It is interesting to observe as the peptide high affinity site for MMP-2 appears to be topologically distinct from the active site, envisaging an allosteric modulation elicited by the α-DG (613–651) peptide on MMP-2, which reduces its enzymatic activity through both a decrease of substrate affinity for the catalytic center (as from α = 1.7±0.4, see Table 2 and Fig 6B) and a slowing down of the rate-limiting step along the pathway of the substrate proteolytic cleavage (as from β = 0.27±0.04, see Table 2 and Fig 6A). Further addition of peptide enables its interaction with MMP-2 active site cleft, thereby exerting a competitive inhibition of the substrate enzymatic processing, as indicated by the lack of an effect on *k*_*cat*_ (see Figs 4B, 4D and 6A).

**Table 2:** All parameters from scheme employed for the description of the modulatory effect by the *α-DG C-term* peptide on the enzymatic activity of MMP-2 at pH 7.3 and 37°C for the fluorogenic substrate (see Fig 5).

	whole MMP-2	cd-MMP-2
^***0***^***k***_***cat***_ **(s**^**-1**^**)**	29.6±4.5	26.1±4.1
^***0***^***K***_***m***_ **(M)**	1.5(±0.3)×10^−6^	1.9(±0.5)×10^−6^
***K***_***a***_ **(M)**	3.5(±0.5)×10^−8^	3.5(±0.5)×10^−8^
***K***_***b***_ **(M)**	2.1(±0.4)×10^−7^	2.1(±0.4)×10^−7^
***α***	1.7±0.4	1.25±0.25
***β***	0.27±0.04	0.32±0.05

It can be observed that the catalytic parameters for the fluorogenic substrate are closely similar (within the experimental errors) between the whole MMP-2 and cdMMP-2 (see Table 2 and Figs 4 and 6). The comparison between the inhibitory mechanism of the peptide operating on the whole MMP-2 and on the cdMMP-2 reveals that for both species the α-DG (613–651) peptide binding sites are located at the catalytic domain and apparently share the same dissociation constants (*i*.*e*., *K*_*a*_ and *K*_*b*_, see Table 2). MMP-2 and cdMMP-2 slightly differ only for the interaction parameters *α* and *β* (see Table 2), likely reflecting small structural differences referrable to the presence or absence of the hemopexin-like domain.

### A dampened activity of MMP-2 towards the α-DG (483–628) domain can be achieved by saturating just one of the two binding sites

**Fig 7:**
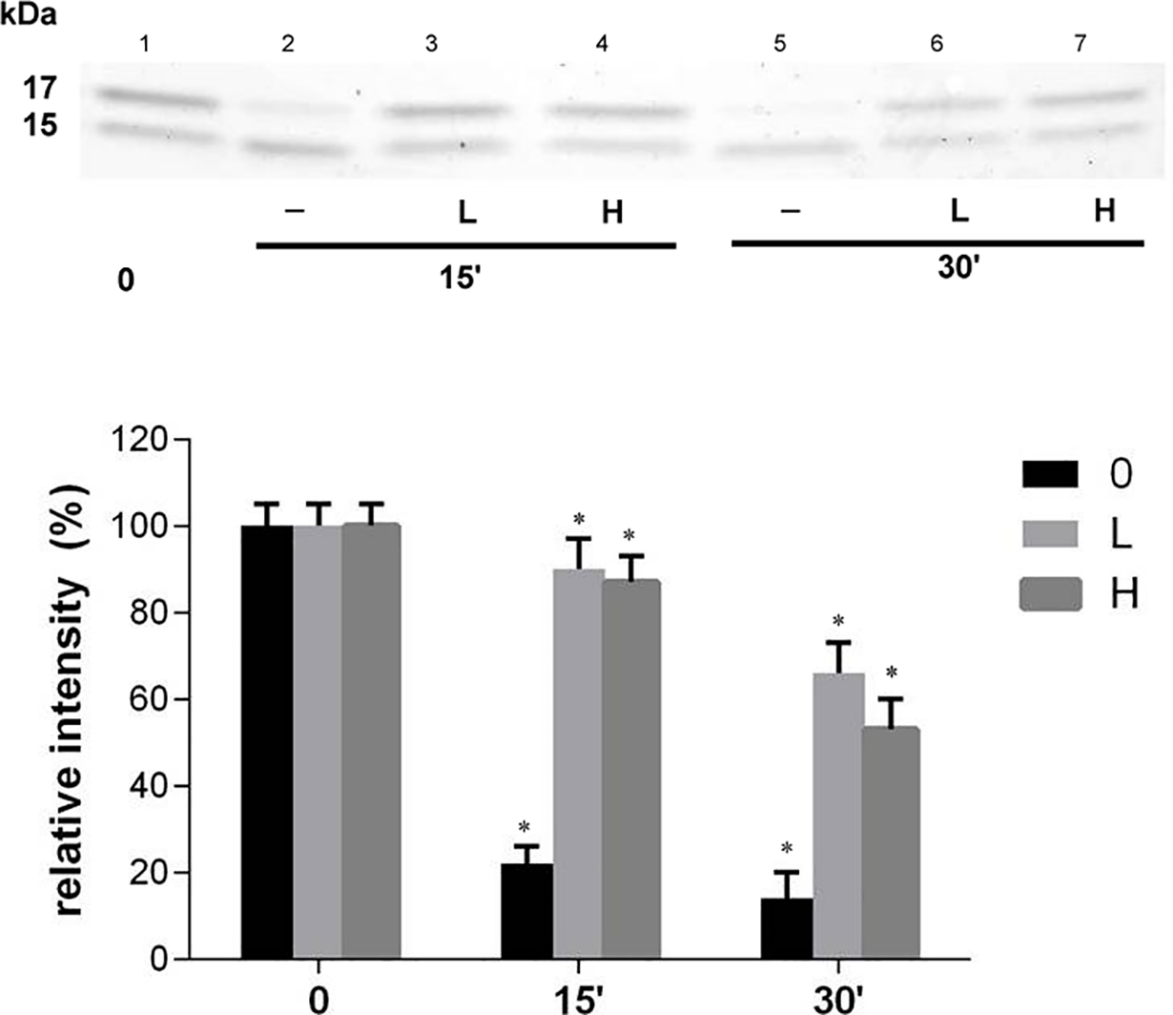
The αDG (613–651) peptide inhibition of the proteolysis of the αDG (483–628) domain by MMP-2. A) SDS-PAGE gel Comassie blue stained showing the MMP-2 processing of 17 kDa band (α-DG C-terminal domain (483–628)) in the presence of none (0), low (L) and high (H) concentration of the *α-DG C-term* peptide (615–651) (0.5 and 30 μM, respectively). A) Three-points time course: time 0 (lane 1), time 15’ (lane 2, 3 and 4), time 30’ (lane 5, 6 and 7). Control points (0): samples in the absence of *α-DG C-term* peptide (lane 1, 2 and 5). B) The amounts of intact α-DG(483–628) domain were densitometry quantified; the histograms reported the relative means±SD band intensity of the 17 kDa band from three independent experiments. A one-way analysis of variance (ANOVA) was performed and followed by Tukey’s honestly significant difference test. (n = 9 for each experimental conditions). (*) represent data significantly different from the respective control (0) at p, 0.05.

To functionally discriminate the effect of each binding site, on the basis of the estimated affinity constants of the α-DG (613–651) peptide for the two sites (i.e: *K*_*a*_ = 3.5(±0.5)×10^−8^ M and *K*_*b*_ = 2.1(±0.4)×10^−7^ M), the inhibition of the α-DG (483–628) domain processing has been compared at two different concentrations of the peptide (*i*.*e*., 0.5 μM and 30 μM). These experimental conditions ensure that the α-DG (613–651) peptide saturates only the allosteric site at the lowest concentration, whereas both binding sites are occupied at the highest concentration. Fig 7 displays the SDS-PAGE comparative analysis of the α-DG (483–628) domain digestion by MMP-2 over the first 30 minutes, clearly showing that in the absence of inhibitor the 17 kDa band almost disappears (15±5% left over) (Fig 7, lane 1 and lane 2). The densitometry analysis shows that the degree of inhibition exerted is very similar (Fig 7, lane 3 and lane 4), despite the two concentrations of the α-DG (613–651) peptide differ by over 50 folds. Within the first 15 minutes of proteolysis, the intensity of the 17 kDa band decreases by only 11±5% under either low or high α-DG (613–651) peptide concentrations (Fig 7, lane 6 and lane 7, respectively). Thus, the inhibition of MMP-2 occurs even when only the allosteric site, but not the active site, is occupied by the α-DG (613–651) peptide. It clearly confirms that the observed inhibitory effect by the α-DG (613–651) peptide is already exerted through the interaction with the allosteric site even when the MMP-2 active site is available for substrate interaction.

### Testing the proteolytic susceptibility of the α-DG (613–651) peptide to MMP-2 by mass spectrometry analysis

Since human α-DG(613–651) peptide is a misfolded sequence of a physiological MMP-2 substrate and one of the sites is able to bind the active site cleft (as indicated by the competitive inhibition behavior, see Fig 4B), we have also evaluated whether MMP-2 can proteolytically digest the α-DG (613–651) peptide. Therefore, we have maximized the chances of proteolysis, not only by prolonging the incubation time (up to 18 hours), but also by increasing both enzyme and peptide concentrations, respectively (*i*.*e*., 600 nM MMP-2 and 150 μM peptide).

**Fig 8:**
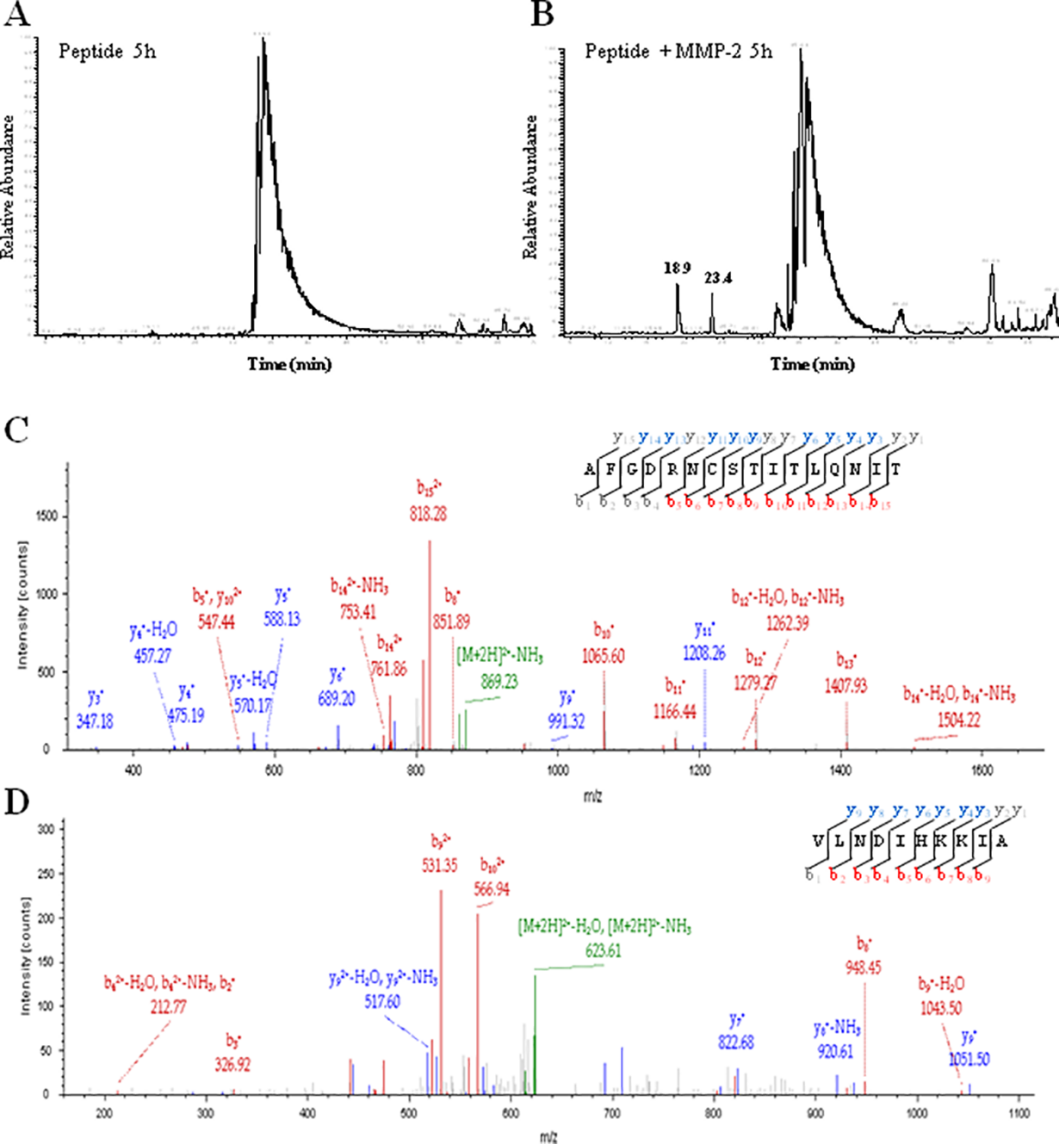
LC-MS/MS analysis of the (613–651) peptide incubated with MMP-2. Chromatographic profiles of the (613–651) peptide incubated for 5 hours without (**A**) or with (**B**) MMP-2. MS/MS spectra of the (636–651) peptide eluting at RT 18.9 min (**C**) and (619–629) peptide eluting at RT 23.4 min (**D**). Matched b and y ions are colored in red and blue, respectively, while precursor ions loosing H_2_O or NH_3_ are indicated in green.

LC-MS/MS analysis of α-DG (613–651) peptide, incubated with MMP-2, revealed the presence of new chromatographic peaks after a 5 hours incubation (18.9 min and 23.4 min retention times), not detected in the absence of the enzyme (Fig 8). Such peaks contain fragments deriving from the α-DG (613–651) peptide processed by MMP-2. In particular, we identified the sequences (619–629) and (636–651) as the most relevant proteolytic products (Fig 8).

These data showed that α-DG(613–651) peptide can be cleaved by MMP-2, although kinetic experiments indicated that it can allosterically inhibit the MMP-2 enzymatic activity on both α-DG(483–628) domain and the fluorogenic peptide. Altogether, these data suggest that the α-DG (613–651) peptide can be cleaved under extreme conditions, such as very high concentrations of both substrate and enzyme (600 nM MMP-2 active enzyme and 150 μM α-DG (613–651) peptide) and prolonged incubation time, while the α-DG(613–651) peptide remains essentially stable under conditions employed for all kinetic experiments performed.

## Discussion

In a previous paper we have shown that MMP-2 is able to enzymatically process the C-terminal domain (corresponding to residues 483–628) of the murine α-DG, whereas a slightly shortened co-purified fragment (now characterized as α-DG(483–621), see Fig 1) could not be degraded, suggesting that the 621–628 a.a. region is likely to include the sequence recognized by the enzyme [35]. This mechanism finds support in the evidence, obtained in this work, that a synthetic peptide, spanning the amino acids 613–651 of human α-DG (which has a sequence highly homologous to murine DG) and partially overlapping with the C-terminus of murine α-DG (Fig 3), is able to efficiently inhibit the enzymatic activity of MMP-2. Interestingly, the α-DG (613–651) peptide also inhibits the α-DG (483–628) domain proteolysis catalyzed by cdMMP-2, which can occur independently on the presence or not of the 621–628 a.a. region, disrupting both the entire protein construct and its fragmentation product. Therefore, the hemopexin-like domain indeed seems to play a role in substrate recognition, since, when it does not assist the catalytic domain, induces a loss of the interaction specificity, making the enzymatic fragmentation of α-DG(483–621) possible (see Fig 2B, *upper panel*).

The α-DG(613–651) peptide turned out to be an efficient allosteric inhibitor of MMP-2 even for the proteolysis on a small synthetic fluorogenic substrate, displaying a bimodal behaviour referrable to both a non-competitive inhibition (characterized by a higher affinity site with *K*_*a*_ = 3.5(±0.5)×10^-8^M, see Table 2) and a competitive inhibition (characterized by a lower affinity site with *K*_*b*_ = 2.1(±0.4)×10^-7^M, see Table 2). Therefore, based on our kinetic dissection, the peptide appears to have two binding sites on the catalytic domain of MMP-2, a first one with a higher affinity and topologically distinct from the catalytic site, and a second one, corresponding to the active site and displaying a lower affinity, where the synthetic peptide competes with the substrate. A comparative analysis of the degradation kinetics of the small fluorogenic peptide carried out by cdMMP-2 (where the hemopexin-like domain has been recombinantly removed) in the presence of the α-DG(613–651) peptide revealed that the MMP-2 and cdMMP-2 share both the high and low affinity binding sites for the inhibitory peptide, with very similar dissociation constants, *K*_*a*_ and *K*_*b*_ (see Table 2). The allosteric modulation of MMP proteolytic function exerted by exosites on the surface of the catalytic domain has already been reported for others member of the MMP family [19]. Therefore, it is reasonable to hypothesize that the binding sites are limited to the catalytic domain. However, our data do not allow to rule out the possibility that the α-DG(613–651) peptide also interacts with the hemopexin-like domain of whole MMP-2, interfering with its binding to the 621–628 amino acidic region.

Due to the close similarity between the amino acid sequence of human α-DG(613–651) peptide and that of the murine C-terminal α-DG(483–628) domain, we can envisage a scenario in which it is likely that the whole MMP-2 binds the α-DG(483–628) domain with very high affinity through the hemopexin-like domain. Binding to this first anchoring site (likely shaped by the 621–628 amino acide sequence) allows MMP-2 to direct its proteolytic cleavage activity on a portion of the dystroglycan C-terminal domain comprised between amino acid positions 483 and 621. On the other hand, the absence of this anchoring site (as in the case of the 15 kDa fragment, see Fig 2A (*upper lanes*) or also in the presence of the α-DG (613–651) peptide) impairs the enzymatic action of MMP-2 on the α-DG (483–628) domain (see Figs 2 and 7).

However, beside this anchoring site for the hemopexin-like domain of MMP-2 on the α-DG 621–628 α-DG domain, an additional site (distinct from the active site) is present in MMP-2, which interacts with the α-DG(483–621) domain. This additional non-competitive site, which is characterized by a high affinity for the α-DG (613–651) peptide, is important for the substrate recognition also in the absence of the hemopexin-like domain, as indicated by the inhibitory effect elicited by the α-DG (613–651) peptide also on cd-MMP-2 (Fig 2B, *lower lanes*). This additional site is likely located at the catalytic domain, possibly involving the CBD domain of MMP-2, which predominantly contributes to substrate recognition by MMP-2 [40, 24].

**Fig 9:**
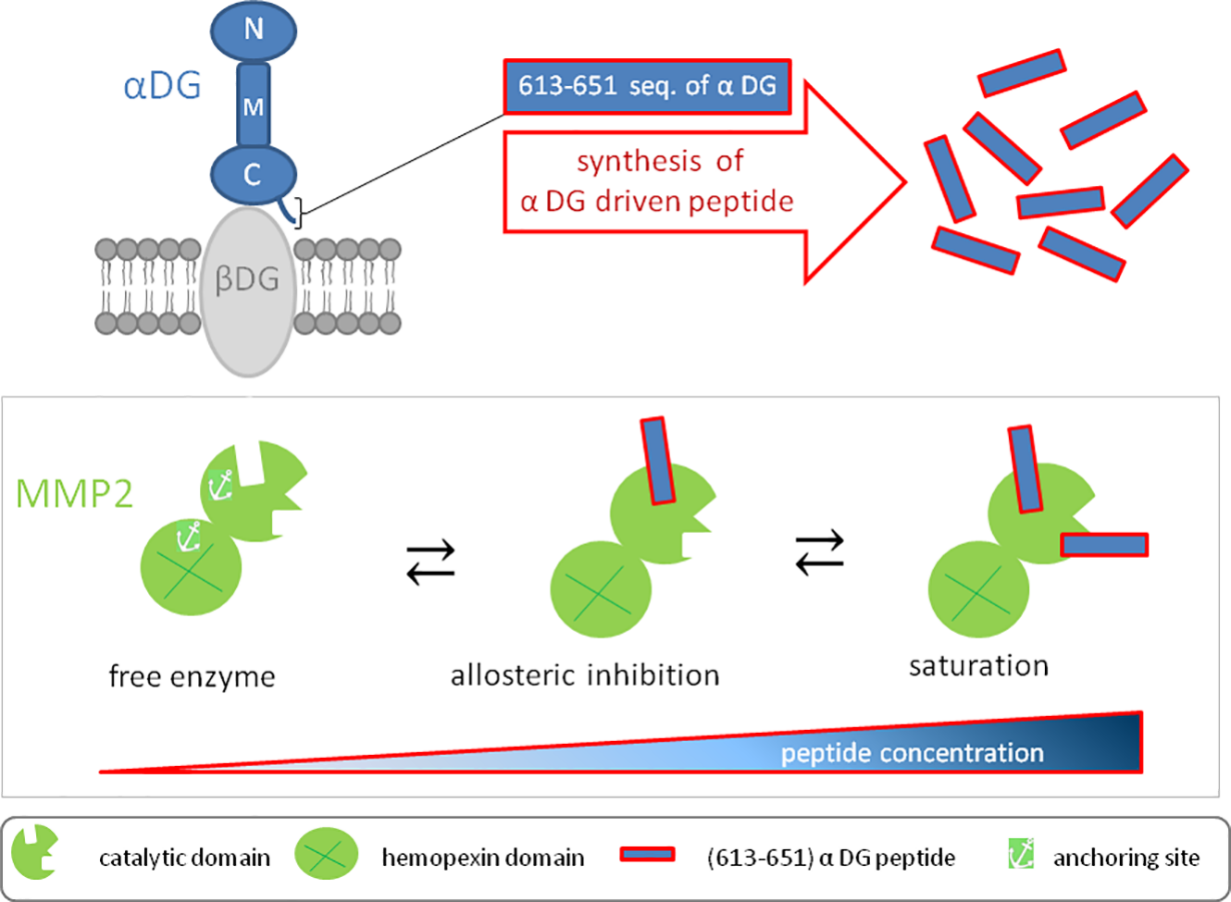
Graphical sketch of the catalytic modulation of MMP-2 by αDG (613–651) peptide. The 613–651 sequence of the human α-DG was used as template to chemically synthesize an MMP-2 inhibitor. This small peptide turned out to modulate the catalysis of human MMP-2 mostly by the two binding sites at the catalytic domain.

As a whole, in MMP-2 two interactions sites (both distinct from the active site) seem to be operative in the recognition of the α-DG (483–628) domain, one at the hemopexin-like domain and one at the catalytic domain. The α-DG (613–651) peptide seems to be able to efficiently impair their action already at a fairly low concentration; under these circumstances, an allosteric inhibition occurs as the peptide does not prevent the proteolytic action of MMP-2 on additional substrates (e.g. fluorogenic substrate proteolysis, see Figs 4B and 9). This feature, which permits to modulate its inhibitory effect, together with the high kinetic stability of the α-DG(613–651) peptide towards the catalytic activity of MMP-2, makes this dystroglycan-derived synthetic peptide a “lead compound”, which may become a potential template for further pharmaceutical development.
